# Long-term medication for ADHD (LMA) trial: 2-year prospective observational study in children and adolescents. Core symptoms, daily functioning, and comorbidity outcomes

**DOI:** 10.1007/s00406-023-01744-1

**Published:** 2024-01-27

**Authors:** M. Johnson, J. Åsberg Johnels, S. Östlund, K. Jakobsson, J. Högstedt, P. Javid Larsson, C. Gillberg, E. Billstedt

**Affiliations:** 1 Gillberg Neuropsychiatry Centre, Sahlgrenska Academy, University of Gothenburg, Gothenburg, Sweden; 2 Habilitation and Health, Region Västra Götaland, Gothenburg, Sweden

**Keywords:** ADHD, Autism, Long-term medication, Daily functioning, Comorbidities

## Abstract

More knowledge is needed about long-term ADHD medication and symptom, daily functioning, comorbidity, and tolerability outcomes. This “Long-term Medication for ADHD (LMA) trial” was a prospective observational 2-year trial in children and adolescents aged 6–18 years (extension of 1-year trial). Participants met criteria for DSM-5 ADHD (inattentive or combined), with complex comorbidities; autism spectrum disorder (31%), autistic traits (24%), oppositional symptoms (59%), anxiety (32%), dyslexia/language disorder (16%), borderline intellectual functioning (17%). Medication was individually tailored and followed-up at clinical visits (1, 2, 3, 6, 12, 18, 24 months). Primary outcome: Clinical Global Impression-Severity and Improvement scales (CGI-S, CGI-I). Secondary outcomes: Investigator-rated ADHD-Rating Scale, Weiss Functional Impairment Rating Scale-Parent report* (*WFIRS-P; Family, School Learning and Behavior, Life Skills, Self-Concept, Social Activities, and Risky Activities domains), comorbidity symptoms and adverse events (AEs). One hundred twenty-eight participants were enrolled (1-year trial only *n* = 27, LMA trial *n* = 101). Of these 29 (23%) discontinued, mainly due to AEs (*n* = 7), moving (*n* = 7), or no longer needing medication (*n* = 6). Main AEs were poor appetite, low mood, anxiety, irritability, fatigue. Improvements from baseline to 2 years were large in CGI-S (effect size (ES) 2.28), ADHD-RS (ES 2.06), and moderate to large in WFIRS-P (ES total 0.73, learning 0.4, family 0.67). Overall, the trial showed robust and sustained improvements in ADHD symptom severity and daily functioning over a period of 2 years of ADHD medication in children and adolescents with ADHD and complex comorbidities. Most AEs were mild. Comorbidity symptoms were improved after 1 year, particularly oppositional symptoms, depression, and anxiety.

## Introduction

Attention deficit/hyperactivity disorder (ADHD) is a neurodevelopmental disorder [1] with an estimated worldwide prevalence of about 5% [2, 3]. It is associated with impairment in several aspects of life and long-term risks for adverse outcomes in academic, occupational, and social functioning, including increased risks for psychiatric comorbidities, accidents, drug use, and antisocial/criminal behaviors [4, 5]. Comorbidity is very common in ADHD [6–9], for instance autism spectrum disorder (ASD), oppositional defiant disorder (ODD), developmental coordination disorder (DCD), developmental language disorder (DLD), anxiety, and depression. To highlight this co-occurrence/overlap of problems and the early onset of unspecific symptoms that may later be identified as neurodevelopmental disorders, the concept of ESSENCE (Early Symptomatic Syndromes Eliciting Neurodevelopmental Clinical Examinations) was proposed by Gillberg in 2010 [10]. However, knowledge about long-term outcomes of medication for these combined disorders is limited. Numerous trials have documented that ADHD medication effectively reduces core symptoms in the short term [11, 12], but less is known about long-term effects on daily functioning and quality of life. Long-term placebo-controlled trials of ADHD medication are rare and difficult to perform, and adherence is problematic both in randomized controlled trials (RCTs) and open-label trials. For example, the Multimodal Treatment of ADHD (MTA) trial documented substantial treatment effects after 14 months of well-controlled medication [7] but in the following observational period, only 70% of the children were still on medication after 2–3 years [13], and treatment benefits dissipated. A Swedish double-blind placebo-controlled RCT of amphetamine treatment for 15 months showed that positive medication effects on inattention, hyperactivity, and other disruptive behavior problems remained during the entire trial [14]. A randomized placebo-controlled withdrawal trial after more than 2 years of methylphenidate treatment in regular clinical care showed continued effects on core symptoms, although with more modest effect sizes than in short-term RCTs [15].

Some naturalistic follow-up studies have demonstrated benefits of long-term ADHD medication in academic performance and psychiatric comorbidity. A case–control follow-up study from childhood to late adolescence showed modestly improved academic outcomes for the group who had been treated with stimulants for a mean duration of about 5 years [16]. A case–control 10-year follow-up study of boys with ADHD diagnosed in childhood followed-up in young adult years found that prior ADHD medication (mean duration 6 years) was associated with reduced risk for depressive, anxiety, and disruptive disorders, and protected against grade retention [17]. A large cross-sectional study with questionnaires to US high-school seniors (modal age 18 years) showed that early long-term stimulant ADHD medication (initiated before age 10, duration of 6 years or more), compared to starting medication later and for shorter duration, reduced the odds of substance use in adolescence [18]. Systematic reviews of multiple large database and national registry studies in children and adults suggest that ADHD medication reduces risks for multiple negative outcomes, including accidents/injuries, mood disorders, suicidality, criminality, substance use disorders, academic impairments. Periods on medication improved outcomes compared to periods off medication [19, 20]. Thus, a considerable body of research suggests that medication to children and adolescents with ADHD may give several benefits but these may be reduced or lost if medication is discontinued. Investigating treatment safety, a recent large European 2-year controlled study of methylphenidate treatment in children and adolescents with ADHD found that treatment was safe and well tolerated [21].

The comorbidity of ADHD and autism has been increasingly recognized during the last decades and several studies have investigated treatment options for these combined disorders [10, 22, 23]. Some studies have reported lower response rates, more adverse effects, and lower tolerated doses in children with ADHD + autism, while other studies found similar treatment effects and adverse effects in both groups. However, most trials are small, studies directly comparing ADHD medication in children with or without ASD are rare, and long-term data are lacking [24, 25]. A recent Swedish observational prospective 3-month study in 323 children and adolescents with ADHD, directly comparing medication outcomes in an ASD group with a non-ASD group, showed similar treatment effects and side effects in both groups [25]. However, most clinical medication trials in ADHD have been performed in selected populations, excluding patients with comorbidities such as autism, and have focused on ADHD core symptoms. More studies are needed with outcomes such as overall functioning and comorbidity development, which may better reflect benefits and well-being that are important in real life, illustrating a net effect of the treatment.

With a view to provide a comprehensive assessment of long-term effects of ADHD medication, we started (in 2014) a prospective observational 1-year trial of medication in children and adolescents with ADHD (the “Qb-trial”) in which a wide range of outcome measures were included; ADHD core symptoms, daily functioning, comorbidity symptoms, and cognitive functioning (IQ). Results from this 1-year trial have recently been published [26, 27]. In 2016, we extended the trial to 2 years of follow-up (the “Long-term Medication for ADHD in children and adolescents (LMA) trial”) and added measures of functioning in various life domains and of Health-related Quality of Life (HRQL) to obtain more data on overall functioning and well-being in the longer term (the HRQL data will be presented in a forthcoming publication).

## Methods

### Sample of patients

All patients who were invited to participate in the trial had been referred to the Child Neuropsychiatry Clinic (CNC) at the Sahlgrenska University Hospital in Gothenburg, Sweden for detailed neuropsychiatric assessment by experienced teams of child psychiatrists, pediatricians, psychologists, special education teachers, and speech-language pathologists. The diagnoses had been ascertained by comprehensive team assessments prior to the trial, most according to DSM-5, some according to DSM-IV prior to the introduction of DSM-5 in Sweden (these were reassessed using DSM-5 criteria at the trial screening visit). After the team assessments, psychoeducation was provided to all patients and families, and teachers were informed about needs for adaptations and support in school. None of the patients received specific psychological interventions during the trial.

### Participants

Children and adolescents aged 6–18 years, with ADHD of any presentation according to DSM-5 and symptoms and impairment that justified ADHD medication, were invited to participate in the 1-year Qb-trial and later in the 2-year trial extension (LMA trial). Neuropsychiatric diagnoses were established by team diagnostic assessments, intellectual level evaluated by WISC-test and rating of adaptive functioning, and comorbidities also assessed by K-SADS-PL interviews. All study interviews and ratings were performed by experienced investigators trained in previous clinical trials. Interrater reliability was tested by joint ratings prior to the trial. To obtain a sample resembling the one commonly seen in clinical practice, all comorbidities were allowed except intellectual disability, bipolar disorder, conduct disorder, substance use disorder, psychosis, severe autism (level 3) or other severe comorbid or medical conditions which would make participation in the trial unsuitable.

Between March 31, 2014 and June 30, 2020, 150 patients were screened for participation in the trials (Qb-trial only *n* = 33, LMA trial *n* = 117), and 128 (82 boys, 46 girls) were enrolled (Qb-trial only *n* = 27, LMA trial *n* = 101). See Table 1 for baseline participant characteristics. Mean age at baseline was 12.0 years (range 6–18.9). All participants had a DSM-5 diagnosis of ADHD, 94 (73%) with combined presentation and 34 (27%) with inattentive presentation, and only 16 (12%) had no comorbidity. ASD was common (*n* = 40, 31%; 26 of these had DSM-5 autism, 14 had been diagnosed with DSM-IV pervasive developmental disorder (PDD) prior to the introduction of DSM-5, and at screening for the trial, they met most but not all DSM-5 criteria). An additional 31 (24%) participants had subclinical ASD (autistic traits). Other comorbidities were ODD, reading/writing disorder or language disorder, borderline intellectual functioning, DCD, generalized anxiety disorder (GAD), subclinical depression, obsessive compulsive disorder (OCD).

**Table 1:** Baseline characteristics (*n* = 128)

Mean age (range)	12.0 years (6–18.9)
	*n* (%)
Male	82 (64)
Female	46 (36)
ADHD inattentive presentation	34 (27)
ADHD combined presentation	94 (73)
*Comorbidities n (%)*	Whole group
ASD (autism or PDD)	40 (31)
Autistic traits	31 (24)
*ODD*	
Full diagnosis	13 (10)
Subclinical	63 (49)
Dyslexia/language disorder	21 (16)
Borderline intellectual functioning	22 (17)
DCD	12 (9)
Tics UNS	12 (9)
*GAD*	
Full diagnosis	10 (8)
Subclinical	31 (24)
Depression (subclinical)	23 (18)
*OCD*	
Full diagnosis	3 (2)
Subclinical	19 (15)
No comorbidity	16 (12)
Full-scale IQ mean (SD, range)	92.6 (12.86, 58–128)

**Fig. 1:**
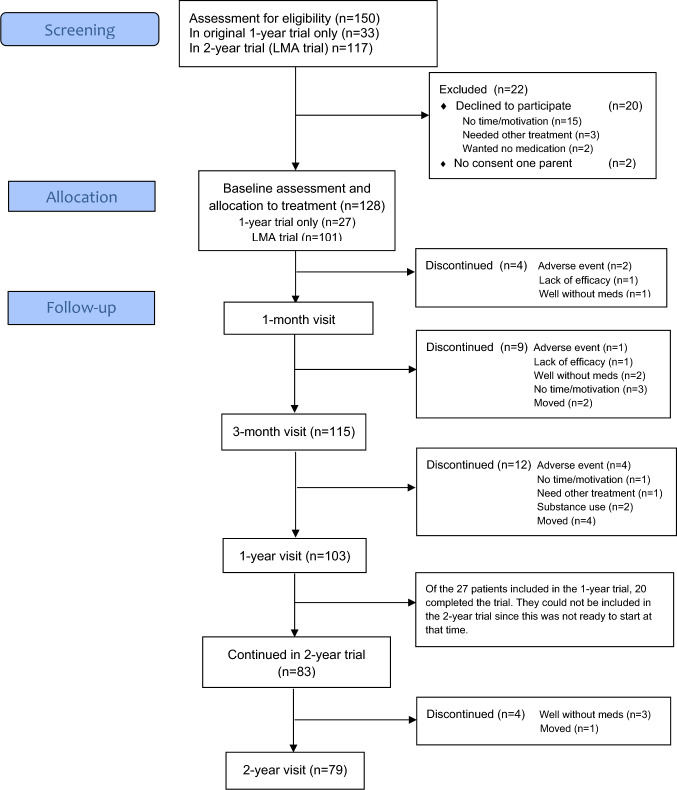
Participant flow chart (modified CONSORT 2010 flow diagram)

Figure 1 shows participant flow during the trials. A total of 128 participants were enrolled in the trials (Qb-trial only *n* = 27, LMA trial *n* = 101). The Qb-trial was completed by 20 (74%) participants and the LMA trial by 79 (78%). Medications initiated at baseline were stimulants (*n* = 117), guanfacine (*n* = 8), or atomoxetine (*n* = 3). A total of 29 (23%) participants discontinued; 25 (20%) during the 1st year (between baseline and visit 5), and another 4 (3%) during the 2nd year (between visits 5 and 7). Reasons for discontinuation were adverse events (*n* = 7), move to another area (*n* = 7), no longer need for medication (*n* = 6), no time/motivation (*n* = 4), lack of efficacy (*n* = 2), substance use (*n* = 2), need for other treatment (*n* = 1). The main adverse events were poor appetite, low mood, anxiety, irritability, fatigue. Twelve participants discontinued or switched their medication (from stimulants *n* = 9, from guanfacine *n* = 3) due to adverse events (two of them discontinued their medication 3–18 months after baseline but remained in the trial unmedicated, and seven discontinued the trial). Including the medication switches, the total number of patients treated at any time in the trials with stimulants were *n* = 117, guanfacine *n* = 15, and atomoxetine *n* = 5. At the end of the LMA trial, 75 of the remaining 79 participants were on any kind of ADHD medication (stimulants *n* = 65, atomoxetine or guanfacine *n* = 10, and 4 patients had discontinued their medication). A few patients had concomitant medication during the trial; selective serotonin reuptake inhibitors—SSRI (*n* = 6 during the whole trial, *n* = 2 during the second year), aripiprazole *n* = 1, risperidone n = 1 (first 4 months), alimemazine *n* = 1 (from 3 months to trial end), hydroxyzine *n* = 1 (whole trial).

### Clinical and functioning assessments

*The CGI-S* scale [28] rates symptom severity and global functioning on a seven-point scale ranging from 1 (normal, not at all ill) to 7 (among the most extremely ill). The *CGI-I* scale rates symptom and functional improvement relative to baseline on a seven-point scale from 1 (very much improved) to 7 (very much worse). Both CGI scales are investigator rated based on all available information from all sources. In this study, treatment responders were defined as patients reaching a CGI-S or CGI-I score of 1 or 2, a definition commonly used in large clinical medication trials [46], indicating a large improvement with only mild symptoms remaining.

*The ADHD-Rating Scale-*IV (ADHD-RS) [29] is an 18-item scale covering all the DSM-IV/DSM-5 ADHD symptoms of inattention, hyperactivity, and impulsivity. In the present trial, the scale was investigator rated. The ADHD-RS is scored on a four-point Likert scale, resulting in a total score and scores on two separate subscales: one of inattention and one of hyperactivity–impulsivity. A higher score reflects greater symptom load. The ADHD-RS is frequently used in medication trials in children and adolescents with ADHD. The scale has been psychometrically validated by the original authors and other researchers [30].

*The WFIRS-P (Weiss Functional Impairment Rating Scale-Parent report)* [31] measures functional impairment during the last month with 50 items covering 6 domains: family (10 items), school learning and behavior (10 items), life skills (10 items), child’s self-concept (3 items), social activities (7 items), risky activities (10 items). It is scored on a 0–3 Likert scale: 0 (never/not at all), 1 (sometimes/a little), 2 (often/much), 3 (very often/very much), or “not applicable” (NA). Lower scores indicate less impairment. Calculating individual mean scores (domain and total scores divided by the number of items) allows direct comparison between domains and excludes items marked NA. In the present trial, we calculated both mean scores and summed scores. Domain or total scores are considered valid if more than 70% or items are scored. Trials in many countries have shown robust psychometric properties for the scale and sensitivity to change during treatment, and it has been used as outcome measure in large clinical trials of ADHD medication in children and adolescents, showing improvement of several aspects of daily functioning, with moderate to weak relation to ADHD symptom measures [32–36]. In a large sample of children and adolescents, a minimally important difference (MID) was defined for WFIRS-P total and domain scores, based on anchor questions to parents indicating that overall problems were at least “a little better” from baseline to follow-up. For the total WFIRS-P score, MID was estimated as a change of 13.47 (ca ½ SD), corresponding to a 0.25 change in total mean score [34, 37]. A large ROC analysis study of WFIRS-P in children with and without ADHD found that a mean total score of 0.65 most accurately discriminates the ADHD group from the non-ADHD group, reflecting a useful cut-off for functional impairment [38].

*The K-SADS-PL (Kiddie Schedule for Affective Disorders and Schizophrenia – Present and Lifetime) 2009 version* [39, 40] is a semi-structured diagnostic interview for children and adolescents aged 6–18 years, based on DSM-IV criteria for several neurodevelopmental and psychiatric disorders, including ADHD, oppositional defiant disorder, ASD, Tics/Tourette syndrome, depression, mania, and anxiety disorders. The K-SADS-PL may be used by the clinician to interview both patients and caregivers. It allows assessment of symptom levels for each DSM-IV criterion (i.e., if clinical, subclinical or no symptoms), and may, thus, indicate if a diagnosis is suspected or not. In this trial, we divided the K-SADS-PL results into three categories; 1—clinical symptom levels suggesting full diagnosis, 2—subclinical symptom levels, and 3—remission, i.e., mild or absent symptoms.

### LMA trial design

The LMA trial was a single-center prospective uncontrolled observational study with clinical visits for screening, baseline, and Visits 1–7 for follow-up at 1, 2, 3, 6, 12, 18, and 24 months. ADHD medication (methylphenidate, amphetamine, atomoxetine or guanfacine) was initiated at baseline according to ordinary clinical practice, tailored to each patient’s individual needs and response, including medication shifts during the trial if needed. The investigators collected data on ADHD symptom severity (ADHD-RS interview), global severity and improvement (CGI-S and CGI-I), adverse events (parent and patient report) and vital signs at all visits. At baseline and Visit 5 (12 months), functional impairments were assessed with the Vineland interview [41], and comorbidities with the investigator-rated K-SADS-PL interview. Daily functioning in various life domains was examined by the parent-rated WFIRS-P Scale at baseline, Visit 5 and 7. HRQL data were collected at the same time points and will be published elsewhere. Cognitive functions were assessed by psychologist Wechsler testing (WISC-IV and WISC-V) [42, 43] at baseline and Visit 5. ADHD symptoms were tested at baseline and Visits 1 and 5 with a computer-based continuous performance test with an additional motion tracking system designed to measure activity (Qbtest) [44]. The 1-year Qbtest, Wechsler, and Vineland data have recently been published [26, 27]. Medication compliance (number of days with doses taken divided by number of days in period) was measured by parent and patient report and was generally high among the participants who remained in the trial (87%, range 52–100%).

### Main outcome measures

The primary outcome of this study was changes in ADHD symptom severity measured by CGI-S and CGI-I during 2 years of well-controlled ADHD medication. Secondary outcomes were changes in ADHD-RS scores, in daily functioning in several domains likely to be impacted by ADHD (Family, School Learning and Behavior, Life Skills, Child’s Self-Concept, Social Activities, and Risky Activities) measured by WFIRS-P, and at the 1-year timepoint the rate and symptom levels of comorbidities assessed by K-SADS-PL interview.

### Statistical analysis

The statistical software SPSS, version 25, was used for all data analysis. Efficacy and safety analyses were performed on the Intention-To-Treat (ITT) population, defined as all participants who had at least a baseline assessment, and on the per-protocol (PP) population (participants who completed all visits) for comparison. Missing data were handled by the last observation carried forward (LOCF) approach. The changes from baseline to endpoints in CGI-S, CGI-I, ADHD-RS-IV, and WFIRS-P scores were assessed with a repeated measures analysis of variance (ANOVA) model. Pre–post baseline vs. Visit 7 effect sizes (ESs), i.e., standardized mean differences (SMD) as per Cohen’s D, and *p* values were calculated. Clinical response rates were analyzed, defined as the proportion of subjects reaching a CGI-S or CGI-I score of 1–2. K-SADS-PL recorded diagnostic status and comorbidities at baseline and 12 months are reported with descriptive statics (numbers and percentages).

## Results

Here we present CGI, ADHD-RS, K-SADS comorbidity, and WFIRS-P data from all patients who had at least baseline ratings. Analysis of HRQL data and outcomes for subgroups with or without comorbidities will be presented in a forthcoming publication.

### CGI and ADHD-RS

**Fig. 2:**
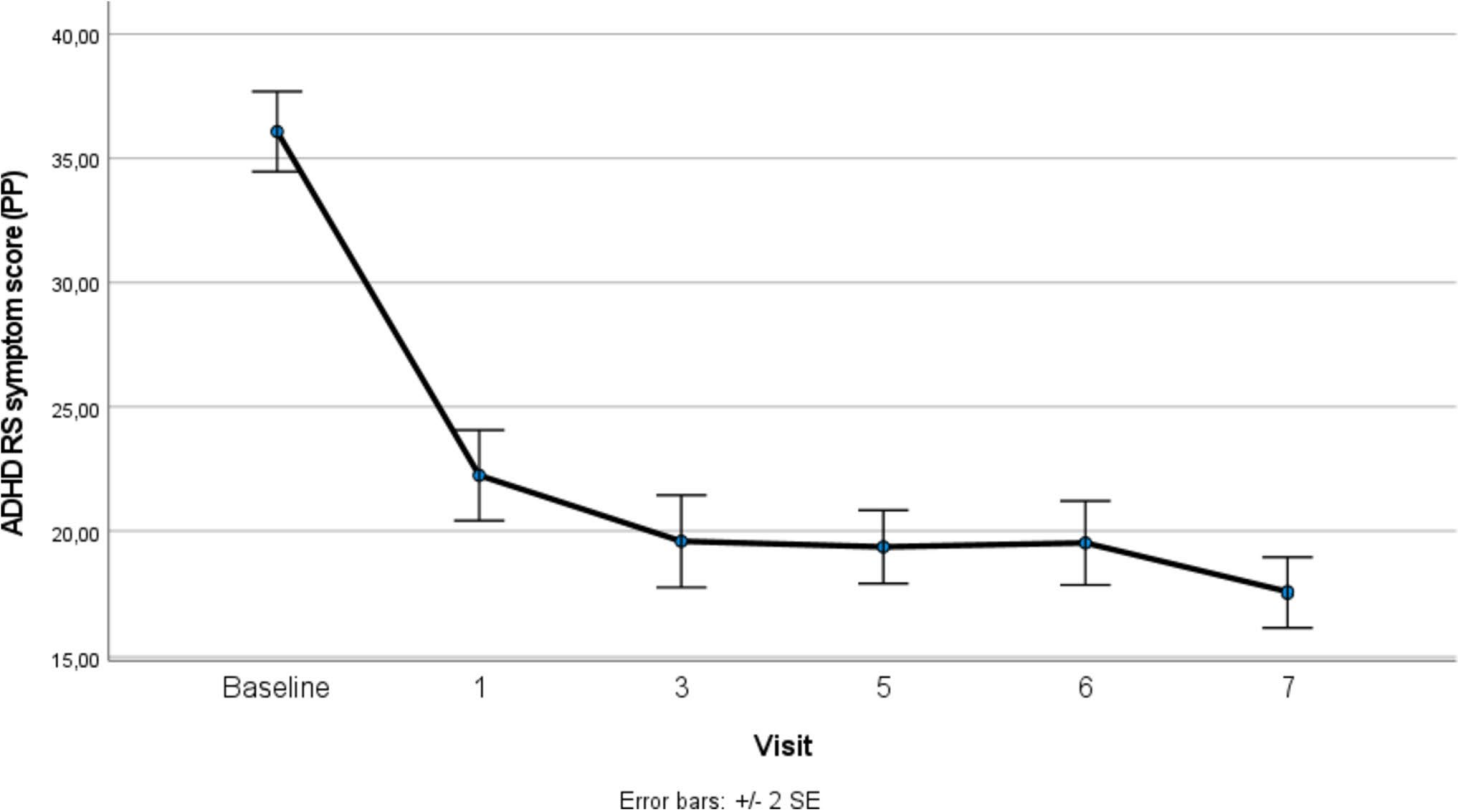
ADHD-RS symptom scores (PP population) across six visits (*p* value from ANOVA < 0.001)

Table 2 shows the changes in ADHD symptom severity according to CGI and ADHD-RS ratings. The baseline mean total score of CGI-S 4.65 and ADHD-RS 35.02 in the ITT (LOCF) population indicated a moderately severe symptom level. Significant improvements were seen at 1 month (Visit 1 mean CGI-S 2.68, ADHD-RS 22.09), and further reductions at 1 year (Visit 5 mean CGI-S 2.00, ADHD-RS 19.88) and 2 years (Visit 7 mean CGI-S 1.99, ES 2.28; ADHD-RS 18.72, ES 2.06). The overall pattern was similar for the ITT (LOCF) and per-protocol (PP) populations, with ES somewhat attenuated in the ITT population. The improvements were significant both in the inattention and hyperactive/impulsive subscales of the ADHD-RS. The clinical response rate, i.e., the proportion of participants in the PP population reaching CGI scores of 1–2 (i.e., CGI-S mild or no symptoms, CGI-I much to very much improved), was 84% (CGI-S)/85% (CGI-I) at Visit 5 (*n* = 101), and 88% (CGI-S)/91% (CGI-I) at Visit 7 (*n* = 79) (Table 2; Fig. 2).

**Table 2:** LMA trial CGI-S, ADHD-RS, and WFIRS-P results for ITT (LOCF) and PP populations

		Baseline M (SD)	Visit 1	Visit 3	Visit 5 M (SD)	Visit 6	Visit 7 M (SD)	F value	ES and significance (baseline vs Visit 7)
CGI-S	PP (*N* = 75)	4.80 (0.55)	2.68 (1.09)	2.17 (0.88)	2.00 (0.90)	1.92 (0.91)	1.65 (0.78)	218.17***	3.50***
	LOCF (*N* = 125)	4.65 (0.73)	2.70 (1.11)	2.36 (1.05)	2.20 (1.06)	2.16 (1.08)	1.99 (1.07)	273.14***	2.28***
ADHD-RS	PP (*N* = 75)	36.08 (6.99)	22.24 (7.90)	19.59 (8.04)	19.36 (6.38)	19.52 (7.33)	17.56 (5.99)	131.46***	2.74***
	LOCF (*n* = 127)	35.02 (7.70)	22.09 (8.37)	20.23 (8.52)	19.88 (7.62)	19.98 (8.14)	18.72 (7.61)	202.58***	2.06***
AD-RS	PP (*N* = 75)	19.33 (3.10)	12.53 (4.89)	10.87 (4.26)	10.55 (3.70)	10.88 (4.45)	10.11 (3.20)	103.26***	2.51***
AD-RS	LOCF (*N* = 127)	18.92 (3.44)	12.28 (4.73)	11.23 (4.39)	10.94 (3.99)	11.09 (4.45)	10.59 (3.85)	175.66***	2.038***
HD-RS	PP (*N* = 75)	16.76 (5.68)	10.00 (4.80)	8.72 (4.85)	8.81 (4.01)	8.69 (3.84)	7.45 (3.85)	93.91***	2.02***
HD-RS	LOCF (*N* = 127)	16.09 (5.90)	9.99 (5.14)	9.02 (5.08)	8.95 (4.62)	8.93 (4.54)	8.14 (4.60)	128.65***	1.53***
*WFIRS-P*									
Total	PP (*N* = 57)	1.05 (0.42)	N/A	N/A	0.75 (0.32)	N/A	0.72 (0.33)	27.46***	0.73***
	LOCF (*N* = 76)	1.03 (0.40)	N/A	N/A	0.80 (0.33)	N/A	0.77 (0.35)	25.56***	0.61***
Family	PP (*N* = 57)	1.18 (0.72)	N/A	N/A	0.86 (0.57)	N/A	0.82 (0.56)	15.69***	0.67***
	LOCF (*N* = 76)	1.20 (0.71)	N/A	N/A	0.95 (0.60)	N/A	0.91 (0.60)	14.96***	0.62***
School learning	PP	2.04 (0.75)	N/A	N/A	1.60 (0.84)	N/A	1.57 (0.86)	12.01***	0.46***
	LOCF	1.96 (0.79)	N/A	N/A	1.62 (0.83)	N/A	1.60 (0.84)	11.25***	0.40***
School behavior	PP	0.76 (0.65)	N/A	N/A	0.39 (0.38)	N/A	0.37 (0.40)	18.73***	0.61***
	LOCF	0.69 (0.63)	N/A	N/A	0.43 (0.42)	N/A	0.41 (0.44)	15.05***	0.47***
Life skills	PP	1.22 (0.58)	N/A	N/A	0.94 (0.48)	N/A	0.91 (0.53)	12.43***	0.56***
	LOCF	1.24 (0.56)	N/A	N/A	1.00 (0.49)	N/A	0.96 (0.53)	14.02***	0.50***
Child’s self-concept	PP	1.35 (0.84)	N/A	N/A	0.84 (0.57)	N/A	0.88 (0.65)	15.08***	0.56***
	LOCF	1.33 (0.81)	N/A	N/A	0.94 (0.63)	N/A	0.96 (0.69)	15.19***	0.49***
Social activities	PP	1.01 (0.68)	N/A	N/A	0.77 (0.53)	N/A	0.66 (0.52)	10.11***	0.52***
	LOCF	0.98 (0.70)	N/A	N/A	0.80 (0.59)	N/A	0.71 (0.60)	9.66***	0.43***
Risky activities	PP	0.48 (0.37)	N/A	N/A	0.26 (0.23)	N/A	0.28 (0.23)	15.25***	0.47***
	LOCF	0.46 (0.34)	N/A	N/A	0.30 (0.24)	N/A	0.31 (0.24)	14.45***	0.40***

*M*   mean, *SD*   standard deviation, *LOCF*   last observation carried forward, *PP*  per protocol. *ES*   effect size (Cohen’s D). ***p < 0.001

Figure 2 presents an illustration of the ADHD-RS changes from baseline to Visit 7, showing that the main improvement occurred between baseline and Visit 1, and was maintained or further improved during the following visits. Normalization of ADHD symptoms (defined as an ADHD-RS score of 18 or less, which means that only mild or no symptoms remained) was attained in 47/101 (46%) of the participants at 1 year and in 48/79 (61%) at 2 years.

#### WFIRS-P

The WFIRS-P also indicated significant improvements over time (Table 2). Here, however, the ESs were mainly in the moderate range. Again, ESs were attenuated in LOCF corrected analyses (ITT population). There were differences between WFIRS-P domains. The highest ES (i.e., the greatest improvement) was reported for Family life functioning, and the lowest for School Learning (Tables 2, 3).

**Table 3:** WFIRS-P summed scores (mean, SD)

Mean (SD)	Baseline	Endpoint (2 years)	Change (%)
*Total*	51.71 (20.22)	34.95 (7.07)	– 16.76 (32%)
*Domains*			
Family	11.97 (14.85)	8.02 (6.36)	– 3.95 (33%)
School learning	7.83 (0.71)	6.05 (0.71)	– 1.78 (23%)
School behavior	4.12 (0.71)	2.16 (0.07)	– 1.96 (48%)
Life skills	12.36 (4.95)	8.84 (0.71)	– 3.52 (28%)
Self-concept	4.0 (3.54)	2.62 (0.71)	– 1,38 (35%)
Social activities	6.84 (9.19)	4.48 (2.12)	– 2.36 (35%)
Risky activities	4.61 (7.07)	2.79 (0.71)	– 1.82 (39%)

*PP* population

Table 3 shows WFIRS-P summed scores at baseline, at the 2-year endpoint (Visit 7), and the score change from baseline to 2 years (PP population). We report these descriptive data to allow comparisons with some prior research that have reported the total (rather than mean) score for the WFIRS-P domains. The total score improved by 32%. The largest domain improvements were seen in School Behavior (48%), Risky Activities (39%), Self-Concept (35%) and Social Activities (35%), and the smallest in the Learning domain (23%).

#### K-SADS-PL

K-SADS-PL interviews were conducted at baseline for 125 participants, of which 108 remained in the study until Visit 5 (1 year) when the endpoint K-SADS-PL interview was performed. Here we present K-SADS outcomes for those who have both baseline and endpoint data (*n* = 96). At baseline, all participants (*n* = 96) had K-SADS scores of ADHD or ADD symptoms at a clinical level. At endpoint, 30 (31%) reached full remission and 66 (69%) scored in the subclinical range (i.e., partial remission). K-SADS remission was a more strict categorical definition than the ADHD-RS score “normalization” described above for 47% of the participants. All the K-SADS remission patients had a CGI-S score of 1 at endpoint = “not at all ill”. For comorbid conditions, the pattern varied depending on diagnosis. Of 20 participants with clinical level of ASD symptoms at baseline, none went into full remission, and only 2 into subclinical/partial remission at endpoint. Of 13 with clinical ODD at baseline, 10 (77%) were improved at endpoint (remission *n* = 2, subclinical *n* = 8). Of 50 with subclinical ODD at baseline, 35 (70%) reached full remission at endpoint and 15 (30%) remained subclinical. No patients had clinical depression at baseline. Of 22 with baseline subclinical depression, 18 (82%) remitted and 4 (18%) remained at endpoint. Of eight with clinical GAD at baseline, four (50%) reached subclinical levels, none remitted fully. Of 23 with subclinical GAD at baseline, 14 (61%) remitted, and 9 (39%) were still subclinical. Of four with tic disorder at baseline, one was subclinical at endpoint, whereas three were unchanged. One patient had clinical OCD at baseline and was unchanged at endpoint. Of 17 with subclinical OCD at baseline, 6 (35%) remitted and 11 remained subclinical. A minority of the participants had two (*n* = 13) or three comorbid full diagnoses (*n* = 4) at baseline. In nine of these participants, these multiple comorbidities were reduced to one (*n* = 7) or no comorbidities (*n* = 2) at endpoint.

### Adverse events and vital signs

Table 4 shows adverse events (AEs) occurring in more than 2% of participants. All AEs were of mild to moderate severity, and most often transient (1–2 months). Twelve participants discontinued or switched their medication due to AEs. Two of them discontinued their medication 3–18 months after baseline but remained in the trial unmedicated. Side effects were all of an expected type based on data from previous clinical trials and clinical experience, and side effect profiles differed between medications. The most common side effects reported with stimulants (*n* = 117) were poor appetite (34%), low mood (21%), insomnia (13%), irritability (12%), anxiety (11%). Mild temporary (1–3 months) weight loss was common in the stimulant group, but prolonged significant weight loss occurred only in (3%) of participants (Table 4). AEs reported with guanfacine (*n* = 15) were fatigue (40%), low mood (20%), irritability, mood swings, and vertigo (13% respectively). Atomoxetine (*n* = 5) was associated with abdominal pain (40%), low mood (25%), irritability, and fatigue (20% respectively). No clinically significant deviations in height, blood pressure, or pulse were observed during the trials (data not shown), with the exception for mild transient tachycardia experienced by six participants with stimulants.

**Table 4:** Adverse events

	AEs occurring in more than 2% of participants		
*n* (%)	Stimulants	Guanfacine	Atomoxetine
	*n* = 117	*n* = 15	*n* = 5
Poor appetite	40 (34)	0	0
Weight loss	4 (3)	0	0
Low mood	24 (21)	3 (20)	1 (25)
Irritability	14 (12)	2 (13)	1 (20)
Mood swings	2 (2)	2 (13)	0
Anxiety	14 (11)	0	0
Insomnia	15 (13)	0	0
Fatigue	1 (0,8)	6 (40)	1 (20)
Headache	6 (5)	0	0
Abdominal pain	5 (4)	1 (7)	2 (40)
Vertigo	0	2 (13)	0

## Discussion

This 2-year open prospective observational trial of ADHD medication in children and adolescents (LMA trial) showed substantial improvements in ADHD symptom severity (CGI and ADHD-RS ratings), daily functioning in several domains (WFIRS-P ratings), and comorbidities (K-SADS-PL assessments after 1 year). To our knowledge, this is the first long-term clinical trial investigating functional outcomes and comorbidity development in patients with ADHD and defined complex comorbidities, particularly autism/PDD or autistic traits. The sample included patients with ADHD combined and predominantly inattentive presentation, but none with predominantly hyperactive/impulsive presentation.

Of 101 patients enrolled in the LMA trial, 79 (78%) completed the whole trial, and the main reasons for discontinuing were AEs, moving to other areas, or no longer needing medication. CGI and ADHD-RS scores showed robustly improved ADHD symptoms already at 1-month follow-up, with further improvements at 1 year and 2 years (Table 2; Fig. 2). Results were similar for the ITT (LOCF) and PP populations, with somewhat lower ESs in the ITT population. The improvements were similarly large both in the inattention and hyperactive/impulsive subscales of the ADHD-RS. At 2 years, clinical response rates (the proportion of participants in the PP population who were much to very much improved, with mild or no symptoms remaining, i.e., CGI-I and CGI-S scores 1–2), were 88% (CGI-S) and 91% (CGI-I). Normalized symptom levels (defined as an ADHD-Rating Scale total score of 18 or less, indicating mild or absent symptoms) were found in 46% of the participants at 1 year and 61% at 2 years. A similar categorical definition was used in the MTA trial to describe treatment success [45].

WFIRS-P total and domain scores had improved at 1 year and were maintained or further improved at 2 years (Tables 2, 3). ESs at 2 years were mainly of moderate size (ITT population 0.40–0.62, PP population 0.46–0.73). Improvements were most evident in the Family and Life Skills domains and in the total score, and smallest in the Social Activities and Risky Activities domains. A recent review of stimulant medication trials showed that the WFIRS-P improvement was strongest in the School (Learning/Behavior) domain, and weakest in the Life Skills, Self-Concept, and Risky Activities domains [34]. These results, thus, show both similarities and differences to our findings, and possible reasons for this may be that the improvement profile is different in the long term, and that our sample is enriched with autism and other comorbidities. A closer comparison to a large placebo-controlled RCT with lisdexamfetamine/methylphenidate (study SPD489-325) [46] provides perspective, although the effect magnitudes are not directly comparable with our trial since ESs in that study were placebo-adjusted. Some ESs in our trial were smaller or similar to those in the 325 study (Family, Risky Activities), some clearly smaller (Total scores, Social Activities), and some clearly larger (Life Skills, Self-Concept). Possible explanations for our larger ES in Life Skills and Self-Concept are that these changes become more developed and visible with longer term treatment, and the smaller ES in Social Activities may be that these are more related to autism, a frequent comorbidity in our sample and according to our K-SADS-PL results less responsive to medication. Another example of a relatively long-term trial that showed significant improvements in the Family, School, Life Skills, and Self-Concept domains was a 6-month open trial with atomoxetine [47].

WFIRS-P optimal scores for discriminating functional impairment from no impairment (both total and domain scores) were calculated in a ROC analysis study [38], comparing children with and without ADHD. The authors concluded that a total mean WFIRS-P score of 0.65 was an optimal cut-off, and that this score level may be useful to define functional remission [34, 38]. The baseline scores in our trial (total and domain) were well above this cut-off (e.g., ITT population total mean score 1.03), indicating considerable impairment. After 2 years, both total and domain scores had improved to lower levels of impairment (total mean score ITT 0.77, PP 0.72). In the Self-Concept (cut-off 1.00) and Social Activities (cut-off 0.71) domains, full functional remission was achieved after 2 years (Self-Concept ITT 0.96, PP 0.88, Social Activities ITT 0.71, PP 0.66). The WFIRS-P total score change in our trial (16.75; 32%) exceeded the MID defined by Hodgkins et al. [37] as a change of 13.47 (ca ½ SD).

Functional outcomes may be seen as real-life net effects of a treatment, and improvements can have important consequences for instance in relations to family and friends, academic and work situations, and for quality of life. These outcomes more closely reflect the treatment goals often expressed by patients than the ADHD symptom improvement itself. WFIRS-P outcomes have been shown to be related to ADHD symptoms, but the functional domains are also partly distinct. ADHD medication may have weaker impact on certain domains, suggesting the need for additional interventions, and it is, therefore, important to document the effects on the patient’s functioning profile [34]. In our study, the response was stronger in the School Behavior subdomain than in the School learning subdomain, suggesting that some learning problems may be more specific (for instance dyslexia/language disorder, dyscalculia, intellectual level) and not entirely associated with the ADHD symptoms, indicating the need for individually tailored special education in addition to the medication.

K-SADS-PL interviews showed that ADHD core symptoms improved from clinical levels at baseline to subclinical levels at 1 year for most participants, and 31% of the patients reached remission. Some comorbidities were substantially improved for many patients (ODD, depression, anxiety), OCD symptoms for some patients, whereas ASD symptoms were improved for a few but unchanged for most. ASD symptoms, thus, seem less responsive to ADHD medication, indicating the need for other interventions and support for autism. No participants reported worsened ADHD symptoms or comorbidities at 1 year. Overall, the K-SADS results indicate an improved behavioral and psychiatric profile for many participants, illustrating increased well-being. Well-being may contribute to improved functioning in several aspects in life, such as social relations, academic functioning, prosocial activities, and reducing risks for maladaptive behaviors. Our findings regarding comorbidities support the results from the case–control study of boys with ADHD diagnosed in childhood followed-up in young adult years [17], which showed association between prior long-term ADHD medication and reduced risks for depressive, anxiety, and disruptive disorders. Our trial extends these results to boys and girls with ADHD, autism, and complex comorbidities.

AEs such as low mood, irritability, mood swings, and anxiety (see Table 4), rarely remained at the 1- and 2-year timepoints. If present, they had often been transient, or had improved on switched medications, or the patients had discontinued the medication.

The compliance to medication in our trial (number of days with doses taken divided by number of days in period) was generally high (87%), and only 12 participants discontinued or switched their medication due to AEs. Development of substance use was rare (*n* = 2/101; 1.9%) and only temporary, suggesting some protective effect of the treatment. This accords with previous research, which has shown that the risk for substance use is several times higher in youth with ADHD, and that an early start of long-term medication reduces such risk [18, 48].

Overall, our results are in line with the large database and national register studies of children with ADHD, which have shown improved academic achievement and reduced risks for several negative outcomes on medication [19, 20]. Thus, the combined evidence from several lines of research suggests that well-controlled ADHD medication may give important benefits in life. However, both register/database studies and clinical trials have reported that benefits can be reduced or lost when medication is discontinued. The long-term MTA trial follow-up for several years after the initial 14 months of well-controlled medication showed that many patients discontinued their medication and treatment benefits were attenuated [13, 49].

The findings in our trial, thus, lend support to the results from earlier research and add data on long-term treatment in a sample of children and adolescents with ADD/ADHD, ASD, and other comorbidities. Future research is recommended with comprehensive functional and quality of life outcomes in long-term trials of patient samples with complex comorbidities similar to the patient samples encountered in clinical practice, to learn more about effects of real-life importance, knowledge that will help clinicians and families to make well-informed decisions about treatment goals.

### Limitations and strengths

The major limitations in the present trial are the open uncontrolled design, and the fact that clinical ADHD ratings were performed by the clinicians involved in the medication interventions. This means that the results may be influenced by other factors than the medication, for instance effects of time and age, positive attitude bias, and the relatively small sample size. Study results mainly reflect the outcome of stimulant medication, since only a few patients took non-stimulants. Strengths of the trial include the long-term follow-up, the prospective observational study design, and the sample of patients with several types of comorbidities, which is the typical situation in daily clinical practice.

## Data Availability

Study data can be made available on personal request from researchers.
